# Senescence regulation by nuclear N-WASP: a role in cancer?

**DOI:** 10.18632/oncoscience.487

**Published:** 2019-08-23

**Authors:** Hui Li, Cord Brakebusch

**Affiliations:** Biotech Research and Innovation Centre (BRIC), University of Copenhagen, Copenhagen, Denmark

**Keywords:** N-WASP, skin cancer, epigenetic regulation

Invasion and metastasis, the hallmarks of malignant cancer cells, are crucially dependent on dynamic changes of the cytoplasmic actin cytoskeleton allowing cell migration. Regulators of the actin cytoskeleton are therefore interesting drug targets to inhibit cancer cell invasion. N-WASP is a ubiquitously expressed promoter of actin polymerization suggested to support cancer cell migration [1, 2]. An intramolecular interaction keeps N-WASP in an inactive conformation, but binding of the Rho GTPase Cdc42 and PIP2 to N-WASP relieves this interaction and opens the conformation of N-WASP and promotes actin polymerisation. N-WASP dependent formation of filamentous actin is triggered by the C-terminal VCA domain of N-WASP and affects endocytosis and filopodia formation. Interestingly, N-WASP is not only present in the cytoplasm, but also in the nucleus, suggesting a role for N-WASP in nuclear actin polymerization or other nuclear processes. Earlier studies indicated that nuclear N-WASP binds to the heat shock transcription factor suppressing HSP90 expression, and to RNA polymerase 2, which might have a regulatory effect on global protein production [3-5].

We could show now that nuclear N-WASP regulates also senescence in keratinocytes and keratinocyte stem cells and thus influences chemically induced skin tumor formation in mice [6]. Mice with a keratinocyte-restricted deletion of the N-WASP gene show increased senescence marks in skin including increased expression of the cell cycle inhibitor p16Ink4a, decreased expression of the DNA maintenance methyltransferase DNMT1, and increased size and granularity of keratinocytes, particularly of the stem cells. This phenotype corresponded to a high resistance of these mice towards DMBA/TPA induced skin tumor formation, either by reduction of keratinocyte stem cells or by senescence of oncogene expressing keratinocytes.

Under hyperproliferative growth conditions in vitro, primary N-WASP null keratinocytes showed premature senescence and stopped growing after few days in culture, supporting a crucial and cell autonomous role for N-WASP in senescence regulation in keratinocytes.

Mechanistical analysis revealed further that nuclear N-WASP binds to the histone methyltransferases G9a/GLP and to the DNA methyltransferase DNMT1, which inhibits the proteolytic degradation of these epigenetic regulators, leading to reduced levels of G9a/GLP and DNMT1 in N-WASP ko cells [6,7]. Interestingly, DNMT1 itself can bind to G9a/GLP [8], but also to β-catenin [9], a crucial transcription factor for keratinocyte stem cells which is required for the formation and maintenance of DMBA/TPA induced skin tumor [10]. Complex formation of DNMT1 and β-catenin was shown to prevent degradation of both, and loss of nuclear N-WASP might therefore contribute to the decrease in β-catenin reported earlier in the skin of mice with a keratinocyte-specific ko of N-WASP. Interestingly, reduction of β-catenin dependent Wnt signaling can also contribute to senescence [11]. Mice with a ko of G9a in keratinocytes show a drastic reduction of DMBA/TPA induced skin tumors, and keratinocyte-restricted ko DNMT1 strongly decreases keratinocyte stem cells, supporting the suggested mechanism explaining tumor resistance of the N-WASP ko mice (Avgustinova 2018).

Deletion of p53 restored G9a/GLP and DNMT1 levels in vitro and rescued the senescence phenotype of N-WASP ko keratinocytes, indicating that N-WASP is a negative regulator of senescence induction by p53 [6]. It was not tested whether a p53 ko also rescues the in vivo phenotype of N-WASP ko mice, but since p53 ko rescues tumor initiation in G9a ko mice [12] this is highly conceivable.

While wild type N-WASP has a senescence-preventing and tumor promoting function in skin, this is not true for all tissues. Deletion of N-WASP in the intestinal epithelium promotes tumor frequency in small intestine in the APC+/− model, although tumor sizes strongly decreased [13]. Surprisingly, in the colon of these mice tumor numbers decreased. Of note, the colon and not the small intestine is the site of intestinal tumor in humans. N-WASP deletion did not affect tumor formation in the APC+/− Kras G12D model.

Are tissue-specific tumor promoting or suppressing effects of N-WASP reflected in alterations of the N-WASP gene in human cancers? Here, tumor suppressor genes are often either deleted or truncated at various positions, while oncogenes are normally characterized by gene amplification or mutational hotspots suggesting activation. Checking more than 10 000 cancers in The Cancer Genome Atlas or more than 40 000 cancers in curated, non-overlapping studies available through cbioportal.org, the answer is not entirely clear. In general, the N-WASP encoding WASL gene is only rarely altered in cancer, amplifications are more frequent than deletions, and truncations are the minority of the observed mutations, indicating a more oncogenic profile of WASL gene alterations in cancer. Though not very prominent, a mutational hotspot is present at V422 in different gastrointestinal cancers where frameshift mutations result in a loss of the actin polymerization inducing C-terminal VCA domain. This mutational hotspot might indicate a role for actin polymerization or for an activating conformational shift as the VCA domain is involved in the inhibitory intramolecular interaction. Other more frequent mutations are R131Q and R141Q, found in uterine endometrial carcinoma and rectal adenocarcinoma. Both mutations are close to the C-terminal end of the N-terminal WH1 domain, which is mediating protein-protein interactions. Finally, truncation mutations at R131 are found specifically in melanoma. It will be interesting to test whether these mutations will show a tumor promoting constitutive inhibition of p53 induced senescence.

N-WASP is structurally highly similar to WASP, which is expressed only in hematopoietic cells. As N-WASP, also WASP is present in both cytoplasm and nucleus, where it was found to interact with histone modifying proteins [14]. Moreover, WASP was recently reported to be important for homology directed repair [15]. WASP binds to double strand DNA breaks and moves them close to each other with help of actin polymerization, which facilitates their repair. A similar function of nuclear N-WASP in non-hematopoietic cells could be of importance for tumor formation and malignant progression. In anaplastic large cell lymphoma, WASP expression is often reduced and functions as a tumor suppressor by controlling mitogen-activated protein kinase activity via Cdc42, which is increased in the absence of N-WASP [16]. A similar effect has not yet been reported in N-WASP ko cells, but might occur in specific cell types.

These recent findings suggest that nuclear N-WASP has cancer relevant functions and that N-WASP mutations might contribute to the formation of specific tumors. However, many important questions remain to be explored:

First, direct nuclear functions of N-WASP should be demonstrated unambiguously by adding a nuclear export signal to the N-WASP gene, thus preventing or at least decreasing its nuclear localisation.

Secondly, it should be explored how the nuclear functions of N-WASP are regulated. Phosphorylation of N-WASP at different residues is likely to play a major role in the regulation of nuclear N-WASP.

Phosphorylation of N-WASP at tyrosine Y256 was described to promote N-WASP retention in the cytoplasm, which might reduce the nuclear effects of N-WASP. Phosphorylation of N-WASP at serine 480/481 correlates with a shift of N-WASP from the chromatin-bound to the soluble nuclear fraction, suggesting that interaction of N-WASP with chromatin bound proteins such as G9a/GLP is regulated by serine/threonine kinases [7]. This could promote cellular senescence in response to oncogenic activation of certain kinase genes. To what extent Cdc42 and PIP2 binding required for nuclear N-WASP function is not known.

Thirdly, it should be explored which protein domains of N-WASP are involved in the interaction with nuclear proteins and to what extent or actin polymerisation is required.

Fourthly, how are nuclear and cytoplasmic functions of N-WASP linked? Are they activated in parallel by regulation of N-WASP conformation or rather antagonistically by changing the NWASP distribution between nucleus and cytoplasm?

Finally, the role of frequent N-WASP point mutations found in human cancers should be explored in mouse models of the respective tumors.

In conclusion, nuclear N-WASP is an interesting novel player in the prevention and promotion of cancer. As N-WASP is involved in actin polymerization in the cytoplasm as well as epigenetic regulation in the nucleus, it might link these processes allowing coordinated cellular responses via regulation of N-WASP. Future studies clarifying these issues are eagerly awaited!
